# Dermatoscopy of atrophoderma of Pasini and Pierini: A case-based insight

**DOI:** 10.1016/j.jdcr.2025.08.021

**Published:** 2025-09-02

**Authors:** Sunil Jaiswal, Shraddha Uprety, Pratichya Thapa, Prakriti Lamichhane

**Affiliations:** Department of Dermatology, Chitwan Medical College, Bharatpur, Nepal

**Keywords:** atrophoderma of Pasini and Pierini, dermatoscopy

## Clinical presentation

**Fig 1:**
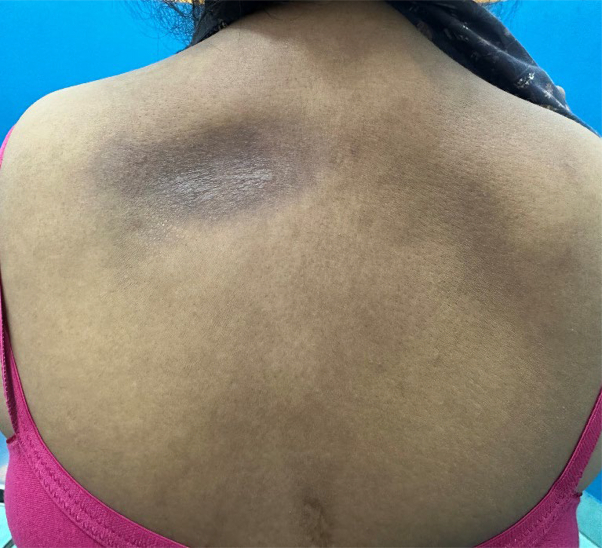
Hyperpigmented nonindurated depressed plaque on the upper back with a cliff-drop border.

A 50-year-old female patient presented with an asymptomatic, solitary, gradually progressive hyperpigmented, nonindurated depressed plaque on the upper back, medial to the left scapula, for 18 years. The plaque was 6 cm in diameter with a sharp edge, giving a cliff-drop border appearance (Fig 1).

## Dermatoscopic appearance

**Fig 2:**
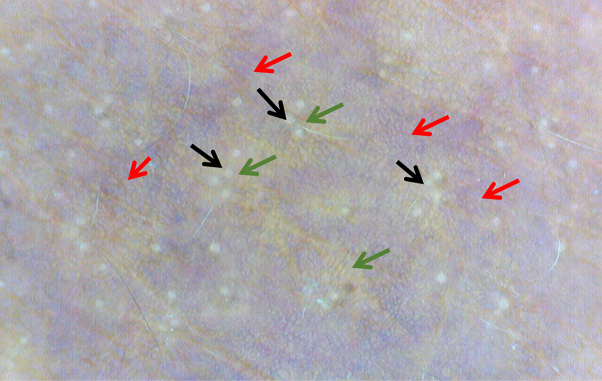
Dermatoscopic image shows violaceous pigment network sparing the follicular region (red arrows), predominant perifollicular brownish pigmented areas (green arrows), and white dots (black arrows).

Dermatoscopy of the lesion revealed violaceous pigmented network sparing the perifollicular region, predominant perifollicular brownish pigmented areas, and white dots (Fig 2).

## Histologic diagnosis

**Fig 3:**
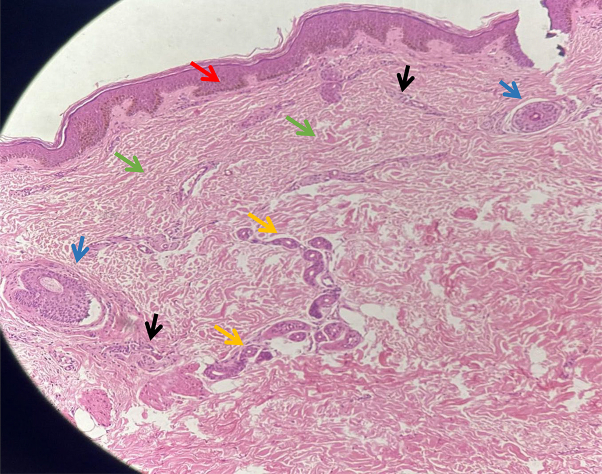
Histopathology (×20) shows epidermis with orthokeratosis, focal acanthosis, and basal layer pigmentation (red arrow). Dermis is fibrocollagenous (green arrow) and shows preserved eccrine glands (yellow arrow) and hair follicles (blue arrow). Mild periappendageal and perivascular lymphocytic infiltration is seen (black arrow).

Histology revealed epidermis with mild orthokeratosis, focal acanthosis, and basal layer pigmentation without atrophy. Dermis is fibrocollagenous with focal thickened collagen bundles, and skin adnexa comprising eccrine glands and hair follicles are also observed (Fig 3).

Key message

Atrophoderma of Pasini and Pierini is an uncommon condition characterized by depressed plaque on the back with a classic cliff-drop edge.^1^ To date, less than 100 cases have been reported in the literature.^2^ Disruption of dermal collagen leads to dermal atrophy in atrophoderma of Pasini and Pierini.^1^ Dermatoscopic findings observed in a few case reports of atrophoderma of Pasini and Pierini are an irregular or regular prominent pigment network, regular perifollicular white areas along with hair preservation, irregularly distributed linear and arborizing blood vessels, and a brownish pigmented area.^3^^,^^4^ In our patient, we observed violaceous pigment network, perifollicular brownish areas with preserved hair, and white dots. The pigment network on dermatoscopy represents a prominent physiological pigment network, whereas perifollicular white areas reflect perifollicular, follicular, and eccrine sparing.^4^ Histopathology remains the gold standard in diagnosing atrophoderma of Pasini and Pierini; however, dermatoscopy can serve as a valuable adjunct in diagnosing as well as differentiating it from morphea.

## Conflicts of interest

None disclosed.
